# Transmission Trees on a Known Pathogen Phylogeny: Enumeration and Sampling

**DOI:** 10.1093/molbev/msz058

**Published:** 2019-03-14

**Authors:** Matthew D Hall, Caroline Colijn

**Affiliations:** 1 Nuffield Department of Medicine, Big Data Institute, Li Ka Shing Centre for Health Information and Discovery, University of Oxford, Oxford, United Kingdom; 2 Department of Mathematics, Simon Fraser University, Burnaby, Canada

**Keywords:** epidemic reconstruction, molecular epidemiology, pathogen genomics, phylogenetics

## Abstract

One approach to the reconstruction of infectious disease transmission trees from pathogen genomic data has been to use a phylogenetic tree, reconstructed from pathogen sequences, and annotate its internal nodes to provide a reconstruction of which host each lineage was in at each point in time. If only one pathogen lineage can be transmitted to a new host (i.e., the transmission bottleneck is complete), this corresponds to partitioning the nodes of the phylogeny into connected regions, each of which represents evolution in an individual host. These partitions define the possible transmission trees that are consistent with a given phylogenetic tree. However, the mathematical properties of the transmission trees given a phylogeny remain largely unexplored. Here, we describe a procedure to calculate the number of possible transmission trees for a given phylogeny, and we then show how to uniformly sample from these transmission trees. The procedure is outlined for situations where one sample is available from each host and trees do not have branch lengths, and we also provide extensions for incomplete sampling, multiple sampling, and the application to time trees in a situation where limits on the period during which each host could have been infected and infectious are known. The sampling algorithm is available as an R package (STraTUS).

## Introduction

The use of genetic data to reconstruct a pathogen transmission tree (a graph representing who infected who in an epidemic) has been the subject of considerable interest in recent years. Many different approaches have been proposed, both phylogenetic (Morelli et al. 2012; Ypma et al. 2013; Didelot et al. 2014; Hall et al. 2015) and nonphylogenetic (Aldrin et al. 2011; Jombart et al. 2014; Skums et al. 2018). In phylogenetic approaches, a phylogenetic tree reconstructed from sequences for pathogens sampled in an epidemic will specify the order of the coalescences of lineages, and also, if its nodes are dated, the time at which these occurred. Some approaches further assume that internal nodes in the phylogeny correspond to transmission events (Morelli et al. 2012; Mollentze et al. 2014; Lau et al. 2015), which in a dated phylogeny specifies infection dates, whereas others do not (Didelot et al. 2014, 2017; Hall et al. 2015; Klinkenberg et al. 2017). In either case, a phylogeny on its own does not determine who infected who, and extra components are required to reconstruct transmission events.

The assumption of coinciding lineage coalescences and transmission events may be unwise, and in particular it does not take into account within-host pathogen diversity (Ypma et al. 2013; Giardina et al. 2017). Several approaches have been taken that do not make it, one of which is to note that if a phylogeny from a completely sampled outbreak has its nodes annotated with the hosts in which each lineage was present, the transmission tree is known (Didelot et al. 2014, 2017; Hall et al. 2015). In particular, Hall et al. (2015) demonstrated that the set of transmission trees for a known phylogeny, with complete sampling and assuming transmission is a complete bottleneck, is equivalent to the set of partitions of its nodes with the property that each part of each partition contains at least one tip and the subgraph induced by the nodes in each part is connected. However, the mathematical properties of this space of partitions remain largely unexplored.

Here, we provide procedures for counting the total number of these partitions (and hence the total number of transmission trees) for a known phylogeny. We also give an algorithm that samples uniformly from the set of such partitions. In a previous paper, Kenah et al. (2016) described a method to perform these procedures when the order of infection times is completely known; here we relax this and no input beyond the phylogeny and a correspondence of hosts to tips is compulsory. Initially we assume that the phylogeny is binary, sampling is complete, each host provided one sample, and nothing is known about the timings of each infection, but we go on in Appendix, Supplementary Material online to relax each of these assumptions individually, and finally relax them all simultaneously.

The procedures outlined here may be useful to researchers wishing to explore the structure that the phylogeny imposes on transmission tree space, or alternatively to explore whether a candidate transmission event is firmly (mathematically) ruled out by a phylogeny or set of phylogenies. Uniform sampling from transmission trees on a phylogeny is rapid and could allow public health researchers who are reconstructing outbreaks a quick guide to some of the most frequently occurring transmission events among all transmission trees consistent with a set of sequence data. We include some numerical applications of our sampling approach, comparing transmission trees on balanced and unbalanced phylogenies, and comparing uniformly sampled transmission trees with transmission trees inferred with the TransPhylo approach (Didelot et al. 2017).

## New Approaches

Here we describe how to count, and uniformly sample, transmission trees for a known phylogeny in the simplest case where the phylogeny is binary, each host in the transmission tree is sampled once and only once, and no time limits are placed on the potential duration of a host’s infectious period.

Let the phylogeny T be an unlabeled rooted binary tree, without branch lengths. Let $T^{*}$ represent the unrooted tree obtained from T by attaching a single extra tip to the root of T by a single edge. Note that two distinct Ts can have the same $T^{*}$, and that $T^{*}$ has one more tip than T.

**Fig. 1.**
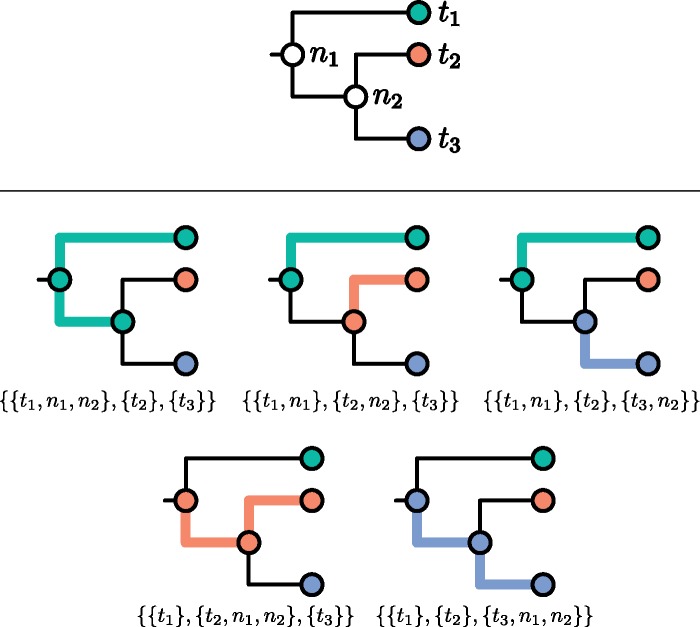
A rooted phylogeny (top) and the five compatible transmission trees labeled with their expression as partitions of its node set (bottom). Thicker, colored branches connect members of the same part.

We follow the correspondence described by Hall et al. (2015) between transmission trees and partitions of the node set of T such that all tips derived from the same host are members of the same part (or block, or subset) of the partition, and the subgraph induced by each part is connected. This assumes that sampling is complete and that transmission is a complete bottleneck (i.e., that only one pathogen is transmitted at a time, so that diversity is not transmitted from host to host). Although we relax the former assumption in Appendix, Supplementary Material online, the latter is more fundamental. See figure 1 for an example. We call a partition that satisfies these constraints an admissible partition.

In this paper, the term “subtree” is intended in the normal phylogenetic (rather than graph theoretic) sense: a subtree is a subgraph of T consisting of a node *u*, all its descendants (if any), and the edges between them. We denote the subtree rooted at *u* by $T_{u}$; this is defined even if *u* is a tip.

### Enumeration of Possible Transmission Trees

With T fixed and having *n* tips, suppose we wish to count the number of admissible partitions, as defined above, of its node set N(T), and hence the set of possible transmission trees. If the set of such partitions is P(T), we wish to calculate |P(T)|. Nothing about the definition of an admissible partition requires a rooted tree, so $P(T^{*})$ is defined similarly. It is trivial that if *n* = 1, then $|P(T)|=|P(T^{*})|=1$. From here on, when we discuss partitions we mean admissible partitions.

If $T_{u}$ is a subtree, we can define $P(T_{u})$ in the obvious way by regarding $T_{u}$ as a tree in its own right. If $T_{u}$ is indeed a subtree in a larger phylogeny of an epidemic, however, this is not sufficient. We do not assume that transmission occurs at the time of internal nodes, and so, even with complete sampling, it is possible that the root node of any subtree was not infecting any of the hosts from which the tips of that subtree were sampled.

To allow for this possibility, we also define a second set of partitions of $N(T_{u}),\;Q(T_{u})$:

$$Q(T_{u})=\left\{\left\{N(T_{u})\cap S:S\in P\}:P\in P(T\right)\right\}$$

An element of $Q(T_{u})$ is the image of an element of P(T) when the intersections of all its parts with the node set of $T_{u}$ are taken. (This is not an injective operation, as the partition of the nodes of T that are not nodes of $T_{u}$ does not matter.)

$Q(T_{u})$, unlike $P(T_{u})$, allows an internal node of $T_{u}$ to share its part with no tip of $T_{u}$. Suppose P is a partition of N(T) and there exists S∈P such that $N(T_{u})\cap S$ is nonempty and contains no tip of $T_{u}$. Then:

1. $u\in N(T_{u})\cap S$ because if it were not then the *S* would not obey the connectedness requirement for being a part of a partition of N(T). This is because, if $v\in S\cap N(T_{u})$ and *t* is the tip of T in *S*, then the path from *v* to *t* must intersect *u*.
2. $N(T_{u})\cap S$ is the only member of the set $\left\{N(T_{u})\cap R:R\in P\right\}$ that contains no tips of $T_{u}$, because u can belong to only one member of a partition of $T_{u}$.

**Fig. 2.**
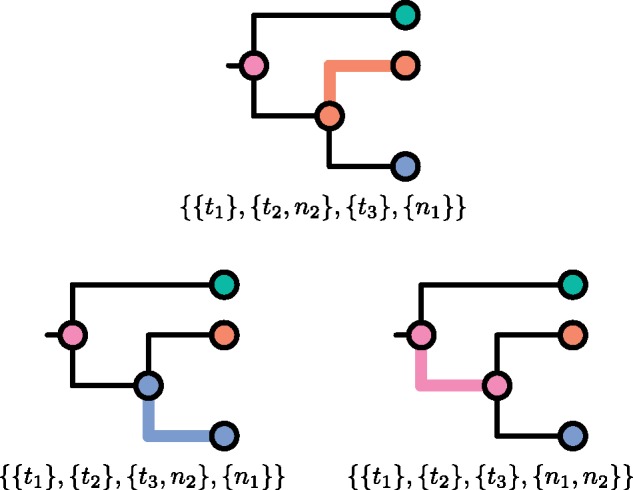
For the tree in figure 1, the three members of Q(T) which are not members of P(T).

It follows that $Q(T_{u})$ is the set of partitions of T which obey the rules for an admissible partition except that they also allow (but do not insist on) an extra part (whose elements still induce a connected subgraph of $T_{u}$) containing T’s root. There is now no need to insist that Q(T) only be defined if T is a subtree of some larger tree; it is defined for any tree. Figure 2 shows an example of the extra elements of Q(T) which are not already elements of P(T) (and hence already displayed in fig. 1).

**Fig. 3.**
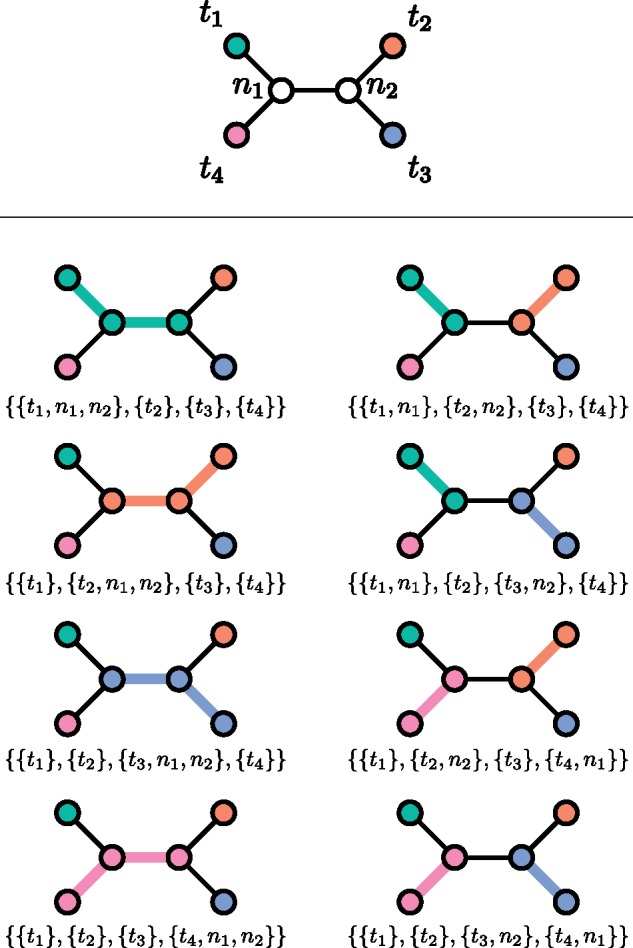
An unrooted phylogeny (top) and the eight partitions of its node set (bottom). Thicker, colored branches connect members of the same part.

We will not need to use the definition of $Q(T_{u})$ again, because it is in obvious correspondence with $P(T^{*})$. (Recall that $T^{*}$ is obtained from T by attaching a single tip to T’s root.) Compare figure 3 with the full set of partitions displayed in figures 1 and 2 as an illustration of this.

If *n* is at least 2, then T has a left subtree $T_{\mathrm{rL}}$ rooted at the left child *rL* of its root node *r* and a right subtree $T_{\mathrm{rR}}$ rooted at the right child *rR*. The following results are proven in the supplementary information, Supplementary Material online:

**Proposition 1.** If T has at least two tips, then

$$\left|P(T)|=(|P(T_{\mathrm{rL}})|\times |P(T_{\mathrm{rR}}^{*})|)+(|P(T_{\mathrm{rR}})|\times |P(T_{\mathrm{rL}}^{*})|\right).$$

**Proposition 2.** If T has at least two tips, then

$$|P(T^{*})|=|P(T)|+(|P(T_{\mathrm{rL}}^{*})|\times |P(T_{\mathrm{rR}}^{*})|).$$

**Fig. 4.**
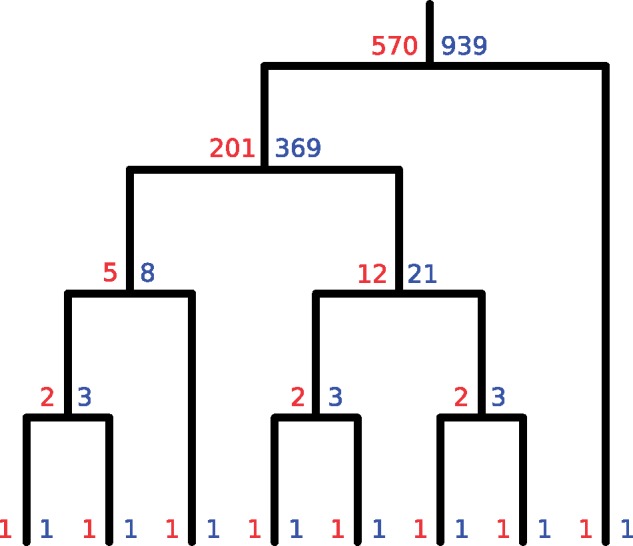
How to count partitions. At each node *u*, if $T_{u}$ is the subtree rooted at *u*, then the red number is $\left|P(T_{u})\right|$ and the blue $\left|P(T_{u}^{*})\right|$. If *u* is internal and has children *uL* and *uR*, $\left|P(T_{u})\right|$ is $\left(|P(T_{\mathrm{uL}})|\times |P(T_{\mathrm{uR}}^{*})|)+(|P(T_{\mathrm{uR}})|\times |P(T_{\mathrm{uL}}^{*})|\right)$ (the sum of the product of the blue number at *uL* and the red number at *uR*, and the product of the blue number at *uR* and the red number at *uL*), whereas $\left|P(T_{u}^{*})\right|$ is $\left|P(T_{u})|+(|P(T_{\mathrm{uL}}^{*})|\times |P(T_{\mathrm{uR}}^{*})|\right)$ (the sum of the red number at *u* and the product of the blue numbers at its children).

Since |P(T)| and $\left|P(T^{*})\right|$ are equal to 1 when T has one tip, |P(T)| can now be calculated for any T by doing a postorder tree traversal, as all that is needed to do the calculations at any node can be obtained by doing the same calculations at both of that node’s children. See figure 4 for an example.

As a mathematical aside:

**Proposition 3.** If $\ell_{n}$ is the fully unbalanced tree (also known as the caterpillar tree) with *n* tips, then $\left|P(\ell_{n})\right|$ is $F_{2n-1}$, the (2n−1)th Fibonacci number, and $\left|P(\ell_{n}^{*})\right|$ is $F_{2n}$.

*Proof* $\ell_{1}$ is the tree with one tip, so $|P(\ell_{1})|=1=F_{1}$ and $|P(\ell_{1}^{*})|=1=F_{2}$, then proceed by induction. For *n* > 1, the two subtrees descended from the root of $\ell_{n}$ are $\ell_{n-1}$ and $\ell_{1}$. $|P(\ell_{n})|=|P(\ell_{n-1})|\times |P(\ell_{1}^{*})|+|P(\ell_{n-1}^{*})|\times |P(\ell_{1})|=F_{2n-3}\times 1+F_{2n-2}\times 1=F_{2n-1}$ and $|P(\ell_{n}^{*})|=|P(\ell_{n})|+|P(\ell_{n-1}^{*})|\times |P(\ell_{1}^{*})|=F_{2n-1}+F_{2n-2}\times 1=F_{2n}.$ □

To give some idea of the size of transmission tree space for a single phylogeny, Proposition 3 shows that $|P(\ell_{10})|=4181,\;|P(\ell_{50})|>2.1\times 10^{20}$ and $|P(\ell_{100})|>1.7\times 10^{41}$.

An alternative, nonrecursive means of calculating both |P(T)| and $\left|P(T^{*})\right|$ using the reduced Laplacian matrix of the wired tree of T (Levine 2009) is given in Section 2 of Appendix, Supplementary Material online. This procedure is less generally applicable as it does not easily extend to incomplete or multiple sampling, but it provides a link to graph theory which may inspire further theoretical work.

### Enumeration of Partitions with a Known Root Part

Having demonstrated how to count the set of partitions or transmission trees compatible with a given T, we now turn our attention to the matter of providing a uniform sample from that set. In order to do this, we need to determine what proportion of the |P(T)| partitions have the root *r* of T sharing its part with each tip.

If E(T) is the tip set of T, and C(T) the set of children of *r*, let a:P(E(T))→P(C(T)) (with P(S) representing the power set of *S*) be the function taking a set of tips of T to the set of children of *r* which are ancestors of (or equal to) at least one of those tips.

Let $\left\{t_{1},\ldots ,t_{n}\right\}$ be the tips of T. For each *i* let *H_i_* be the set containing just *t_i_*; this may seem redundant but it becomes crucial when relaxing the single sampling assumption as described in Appendix, Supplementary Material online. If $P^{i}(T)\subseteq P(T)$ is the set of partitions of N(T) that have *r* in the same part as the membership of *H_i_*, we wish to calculate $\left|P^{i}(T)\right|$ for all *i*. Naturally $\sum_{1\leq i\leq n}|P^{i}(T)|=|P(T)|$. If T has one tip $t_{1}\in H_{1}$, obviously $|P^{1}(T)|=1$. For any other T, treating $T_{\mathrm{rR}}$ and $T_{\mathrm{rL}}$ as trees in their own right but whose tips are partially shared with T, we can define $P^{i}(T_{\mathrm{rL}})$ (respectively, $P^{i}(T_{\mathrm{rR}})$) only if $a(H_{i})=\{\mathrm{rL}\}$ (respectively, $a(H_{i})=\{\mathrm{rR}\}$). The following is proven in Appendix, Supplementary Material online:

**Proposition 4.** Suppose T has at least two tips. Then:

$$|P^{i}(T)|=\{\begin{array}{cc} |P^{i}(T_{\mathrm{rL}})|\times |P(T_{\mathrm{rR}}^{*})|, & a(H_{i})=\{\mathrm{rL}\}\\ |P^{i}(T_{\mathrm{rR}})|\times |P(T_{\mathrm{rL}}^{*})|, & a(H_{i})=\{\mathrm{rR}\}\\ 0, & a(H_{i})=\emptyset \end{array}.$$

Proposition 4 allows the value of $\left|P^{i}(T)\right|$ for all *i* to be calculated by a similar postorder traversal to that described in the previous section. See supplementary figure S1, Supplementary Material online for an example. Note that with an algorithm to calculate all $\left|P^{i}(T)\right|$ available, a separate one to calculate |P(T)| is not necessary as the latter is simply the sum of the former. As *n* calculations are performed at *n* – 1 nodes, the calculation of $\left|P^{i}(T_{u})\right|$ for all internal nodes *u* of T is $O(n^{2})$; in other words the number of required operations scales quadratically with the number of tips of phylogeny.

### Sampling Uniformly from P(T)

If the postorder traversal above is complete (and its results recorded for all subtrees of T, not merely T itself), sampling a random partition requires a single preorder traversal. We start with a collection of empty sets $P=\{S_{1},\ldots ,S_{n}\}$, where each *S_i_* is to contain the set *H_i_*; once the traversal is complete, P will be a partition of N(T). The traversal starts at *r*, and the $\left|P^{i}(T)\right|$ can be used as a set of probability weights for a draw of the *S_i_* that *r* belongs to, as they determine, for each *i*, how many of the |P(T)| total partitions have *r* sharing a partition with the members of *H_i_*.

Subsequently, when the traversal reaches another node *u* with parent *uP*, and we have already placed *uP* in *S_i_*, then *u* must also be placed in *S_i_* if *t_i_* is one of its descendants (by connectedness) or if *u* is *t_i_* itself. Otherwise, there are $\left|P(T_{u}^{*})\right|$ ways in which $T_{u}$ can be partitioned, since it can be a member of the same part as *uP* or a member of the same part as each of its tips. $\left|P(T_{u}^{*})|-|P(T_{u})\right|$ of these have *u* in the same part as *uP*, whereas the remaining $\left|P(T_{u})\right|$ do not. For each *j* such that $t_{j}\in E(T_{u}),\;|P^{j}(T_{u})|$ gives the numbers of ways in which u can be placed in the same part as *t_j_*. The part for *u* can then be sampled with probability given by a weight vector that has $\left|P^{j}(T_{u})\right|$ for each *S_j_* if $t_{j}\in E(T_{u}),\;|P(T_{u}^{*})|-|P(T_{u})|$ for *S_i_*, and 0 for any other part.

Although the sampling procedure requires a single $O(n^{2})$ calculation to establish the values of each $\left|P^{i}(T)\right|$ and $\left|P^{i}(T^{*})\right|$, the uniform sampler itself is only O(n); its complexity scales linearly with the number of tips of T and large samples can be acquired rapidly.

### Software Implementation

The enumeration and sampling algorithms described above, as well as the extensions described in Section 3 of Appendix, Supplementary Material online, are implemented in an open-source R package entitled Software for Transmission Tree Uniform Sampling (STraTUS), available at http://github.com/mdhall272/STraTUS; last accessed March, 26 2019. There are two key functions in the package. The first is tt.generator, which takes as input an phylogenetic tree produced by, for example, the ape package (Paradis and Schliep 2019), as well as optional arguments specifying the maximum number of unsampled hosts in the transmission chain, upper and lower bounds on infectious periods and assignment of tips to hosts, and calculates the values of $\left|P^{i}(T_{u}^{})\right|$ and $\left|P^{i}(T_{u}^{*})\right|$ for each *i* and *u*. The output of tt.generator can then be given to the second function, sample.tt, in order to generate a uniform sample of transmission trees of any size. Graphical display of the node colorations in the sample (using ggtree; Yu et al. 2017) and representations of the transmission trees as igraph objects are supported.

## Results

Sampling random transmission trees that are consistent with a known phylogenetic tree has applications in transmission inference and in phylodynamics. In particular, there has been some work on whether imbalanced phylogenies are indicative of specific kinds of transmission (Leventhal et al. 2012; Frost and Volz 2013; Robinson et al. 2013; Colijn and Gardy 2014). It is clear that the phylogenetic tree places some constraints on who may have infected whom, particularly if individuals are treated and become uninfectious at the time of sampling. The current work aids investigations of this nature by permitting quantitative comparison of transmission trees sampled uniformly at random from two different phylogenetic trees.

The shapes of phylogeneties have been related to transmission patterns in a number of studies, as phylogenetic data are an appealing alternative to classical methods, such as contact tracing, to investigate transmission particularly in settings where highly transmitting individuals may be difficult to identify directly, for example, in sexually transmitted or blood-borne infections (Leventhal et al. 2012). In particular, how the so-called “superspreaders” (individuals transmitting an infection to a large number of secondary cases), or contact number heterogeneity more broadly, may leave a signature in phylogenetic trees is one important phylodynamic application, particularly in HIV. Several studies have related contact number heterogeneity to the imbalance and cluster patterns in phylogenetic trees, with conclusions that differ depending on assumptions about the network structure and dynamics and the simulation approach (Leventhal et al. 2012; Frost and Volz 2013; Robinson et al. 2013; Colijn and Gardy 2014). One of the most commonly used ways to describe the shapes of phylogenetic trees is with their overall asymmetry (imbalance), via, for example, the Sackin index (Sackin 1972). Indeed, in the phylodynamic literature, this and the number of cherries in the phylogeny have been the primary measures of tree shape. We explored whether there is a systematic difference in the offspring distribution in randomly sampled transmission trees resulting from their asymmetry.

**Fig. 5.**
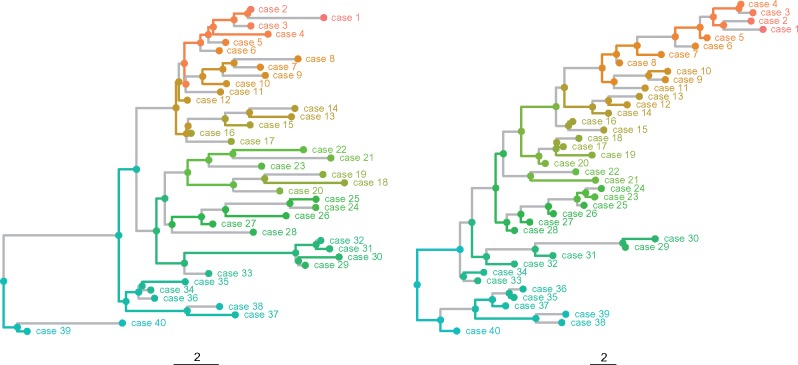
Yule (top) and biased (bottom) phylogenetic trees with randomly sampled partitions. Each color corresponds to a part of each partition. Gray edges separate nodes that are in different parts of the partition. Branch lengths are assumed to be in arbitrary time units.

We began with two input phylogenetic trees each with 40 tips. The phylogenetic topologies were randomly generated using the apTreeshape R package. One tree came from a Yule model (a pure branching process) and the other from a so-called “biased” model with a bias parameter 0.9. The branch lengths for each were then redrawn from a gamma distribution with shape parameter 1.6 and scale parameter 1 to produce phylogenies with the appearance of heterogenous sampling times (if their branch lengths are assumed to be in calendar time). The “biased” model is a growing tree model; the children of a lineage with a speciation rate *r* have rates pr and (1−p)r. This produces imbalanced trees. The two input trees, along with a randomly sampled partition assuming full sampling and only one tip per host, are shown in figure 5.

We sampled 300 transmission trees uniformly at random on our 2 input phylogenetic trees, with full sampling and 1 tip per host, and compared the distribution of offspring, that is, the number of secondary cases infected by a host. Note that this is distinct from the offspring distribution of a speciation process of the type that may be used to generate a phylogeny; in that case speciation events are represented by nodes, an assumption that we do not make. With full sampling, the mean number of secondary cases per source in a tree is just under 1, because each individual except the source has a single infector.

**Fig. 6.**
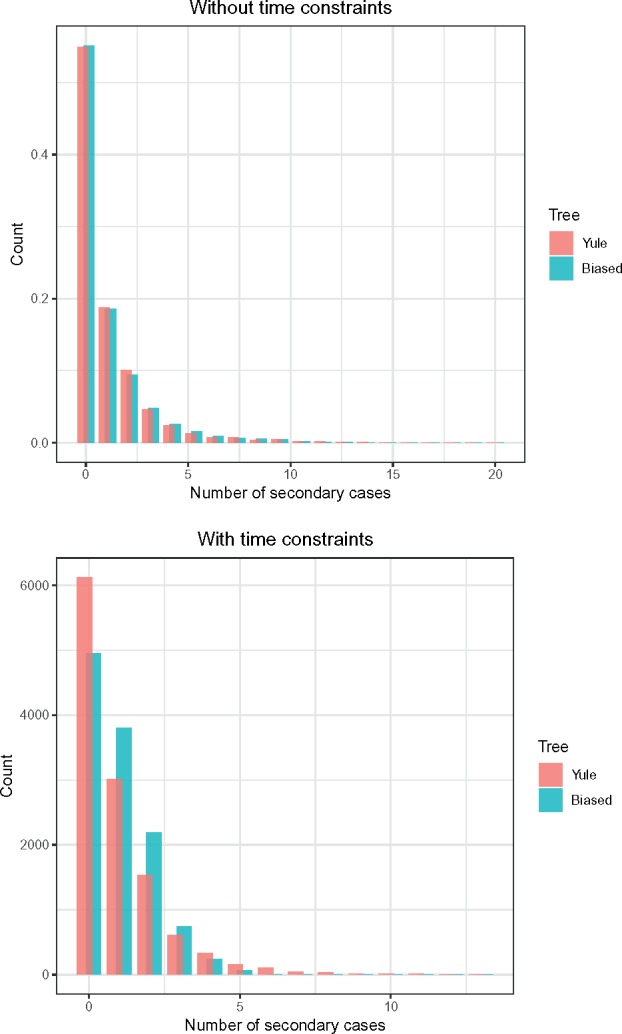
Offspring distributions from two input phyogenetic trees without (top) and with (bottom) constraints on the time between infection and sampling such that hosts became noninfectious immediately upon sampling, and had been infectious for a maximum of 3.5 time units, compared with the mean branch length in these trees of 1.39 and 1.63 time units, respectively.

We find that the relationship between the phylogenetic tree and the dispersion of the offspring distribution depends on whether the timings of infection are restricted. When we make no such restrictions, there is sufficient flexibility in who may infect whom that the two trees have very similar offspring distributions. In contrast, if we constrain the heights of nodes in each tip’s part of the partition according to an infectious period, such that each host becomes noninfectious upon sampling and becomes both infected and infectious no more than 3.5 time units before sampling (using the sampling procedure outlined in Appendix, Supplementary Material online), the transmission tree from the more imbalanced phylogeny has fewer nodes with no children but more with one or two, suggesting a tendency towards sequential transmission compared with more frequent superspreader-like dynamics in the balanced version (fig. 6).

**Fig. 7.**
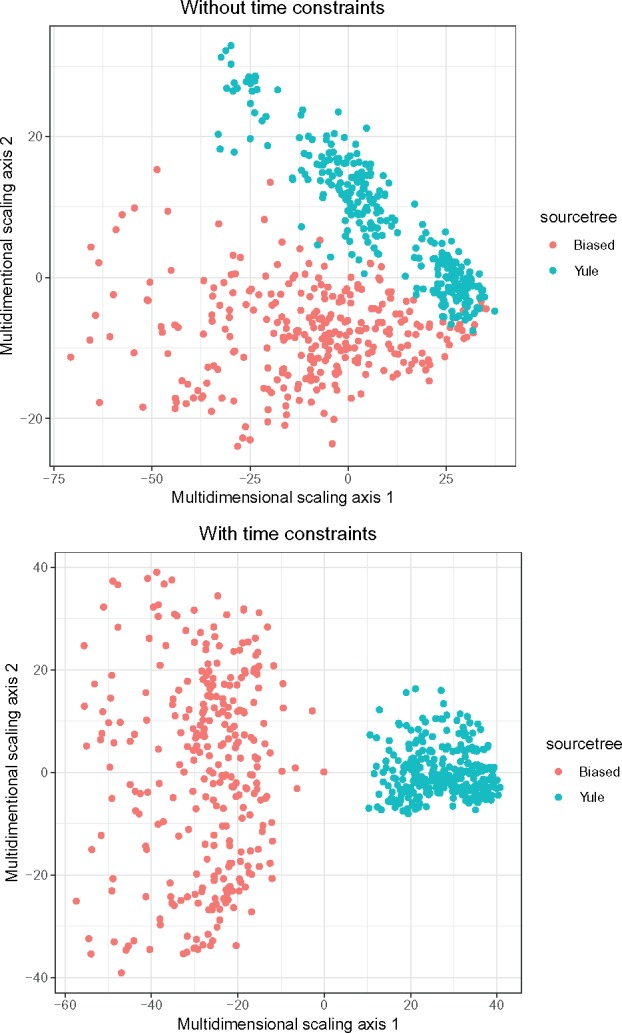
Multidimensional scaling plots visualizing distances between transmission trees sampled on the Yule and biased phylogenies, without and with restrictions on the lengths of infectious periods.

We also compared the transmission trees sampled on the biased and Yule phylogenies directly, using the metric approach developed by Kendall et al. (2018). Briefly, the metric is a distance between two transmission trees; the distance is zero if and only if the transmission trees are the same (except for some sets of unsampled cases which are not relevant here, as we used full sampling). We compute the distances between all pairs of trees, and visualize the distances using multidimensional scaling (MDS). Figure 7 shows the results both with and without time constraints. Without time constraints, the Yule and biased phylogenies both admit a “wide spread” of possible transmission trees, but while there is a small overlap they are for the most part strongly separated on the plots. With time constraints the spread is notably greater for the biased tree, whereas the transmission trees for the two phylogenies form entirely distinct clusters. This is a visual illustration of the fact that the structure of the phylogeny places consistent constraints on admissible transmission trees, and how the imposition of limits on infectious periods differentiates them further.

**Fig. 8.**
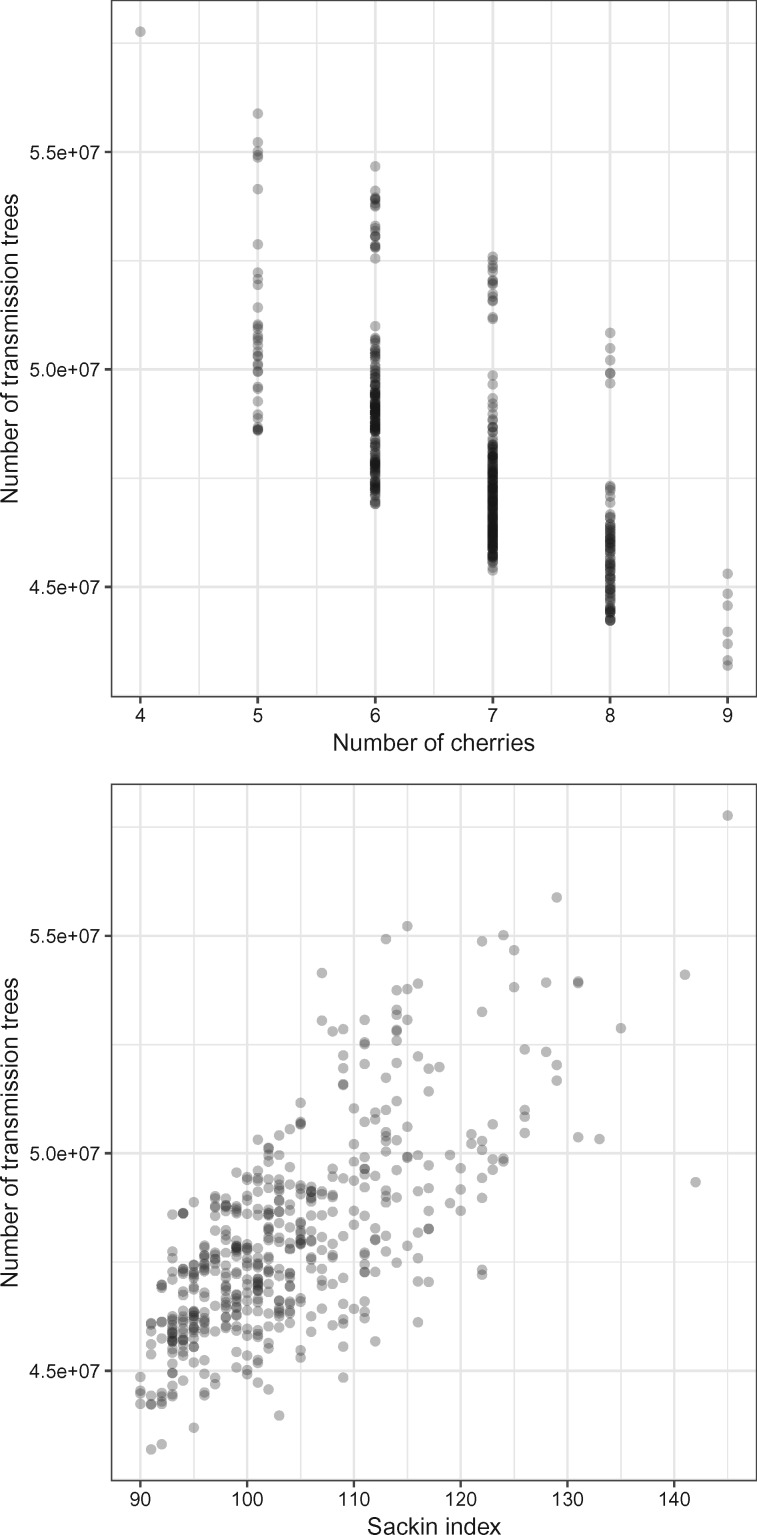
PCA plot illustrating the distances between transmission trees inferred by TransPhylo and sampled using STraTUS, derived from the timed phylogenetic tree of the Roetzer outbreak, previously published by Didelot et al. (2017). The colors indicate the algorithm used and the number of unsampled cases selected in STraTUS. The shaded areas enclose all the trees in each sample and give an idea of the extent of the corresponding MDS spaces. The squares represent the geometric median tree of each sample.

We then sampled 500 random phylogenetic trees of 20 tips each using ape (Paradis and Schliep 2019) and computed the number of transmission trees each one admits. We also computed two common tree shape statistics: the number of cherries and the Sackin imbalance. A cherry is a configuration consisting of two tips and an internal node. Each binary phylogenetic tree with *n* tips has at least one cherry and could have at most n/2 cherries. The Sackin imbalance (Sackin 1972; Blum and François 2005) has been defined in several ways, including the total or alternatively the average path length from a tip to the root of the tree. Broadly (see figure 8), the number of possible transmission trees compatible with a phylogeny increases as the Sackin imbalance of that phylogeny increases, and declines as the number of cherries increases (cherries are symmetric feature, so trees with higher numbers of cherries tend to have a lower Sackin imbalance). This is, again, under the assumption that there no constraints on the timing of transmission relative to the node’s sampling time.

Finally, we compared randomly sampled transmission trees with transmission trees estimated by the TransPhylo algorithm (Didelot et al. 2017). Our aim here is to investigate whether, if sensible constraints on infectious periods are known, the fast uniform sampling approach can yield a comparable set of transmission trees to full statistical model inference using MCMC. We used an outbreak of tuberculosis cases over a 13 year period in Hamburg, Germany, which was previously published (Roetzer et al. 2013) and previously analyzed using TransPhylo (Didelot et al. 2017). Because the current version of STraTUS cannot apply limits on infectious periods to unsampled cases (see Appendix, Supplementary Material online), we applied the two algorithms to a 72-tip subtree in which the root node of the epidemic was plausibly infecting a sampled host (see supplementary fig. S2, Supplementary Material online). (This restriction in STraTUS means it will not favor any particular number of unsampled hosts in the transmission tree along the branches separating the root node from the first sampled case, regardless of the lengths of those branches. This is very different to TransPhylo, so we ensure that the root case was plausibly sampled in order to make a comparison.) We sampled transmission trees uniformly at random with STraTUS, and compared them with the TransPhylo-estimated trees. The timed phylogeny was estimated using BEAS (Suchard et al. 2018) and was the same as reported in Didelot et al. (2017), then pruned to the 72-tip subtree. We restricted the maximum possible time between the point of infection and sampling to 7 years (permitting cases to become infectious immediately upon infection), and assumed that cases become noninfectious upon sampling. We generated multiple STraTUS samples for 0 and 40 unsampled hosts, and also with the unsampled count drawn from the empirical distribution of unsampled hosts from TransPhylo. The median number of unsampled hosts from TransPhylo was 39.

**Fig. 9.**
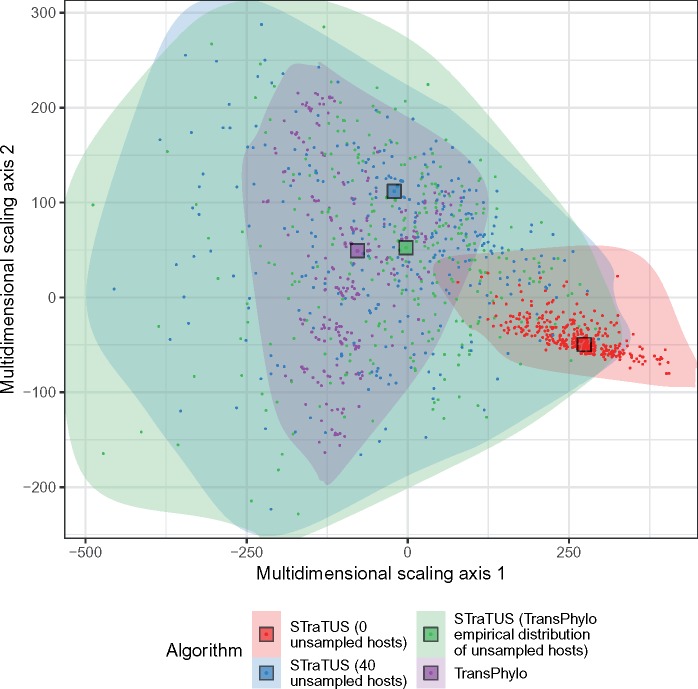
Geometric median trees from TransPhylo (left) and STraTUS (right). Gray nodes represent unsampled cases and in each case the index host in the tree is ringed in black. Although many individual transmission events differ, there are many points where the differences are “minor” and the trees share small subclusters of cases who transmitted to each other in different configurations.

We used the metric and MDS approach outlined above to compare the sets of transmission trees. Figure 9 illustrates the results in 2D MDS. For 40 unsampled hosts and when the unsampled count was drawn from the TransPhylo empirical distribution, the STraTUS sample occupies much of the same space as TransPhylo, but the STraTUS transmission trees are much more widely distributed. This is not surprising, as the sampling of a TransPhylo tree is determined by its posterior probability under a phylodynamic model, whereas STraTUS is a cruder, uniform sample from the space of all admissible phylogenies. The STraTUS sample with no unsampled hosts, on the other hand, forms a largely distinct cluster in the plot from the TransPhylo trees.

We also determined the tree within each group that is closest to the center of the trees (the geometric median tree; Jombart et al. 2017). These are marked in figure 9. Notably, the STraTUS sample whose median is closest to the TransPhylo median is the one where the unsampled host count was drawn from the TransPhylo empirical distribution. These results suggests that it may be possible to use STraTUS to quickly produce an approximate sample of possible transmission trees for a given phylogeny, but that unbiased estimation of the number of unsampled individuals would be necessary.

**Fig. 10.**
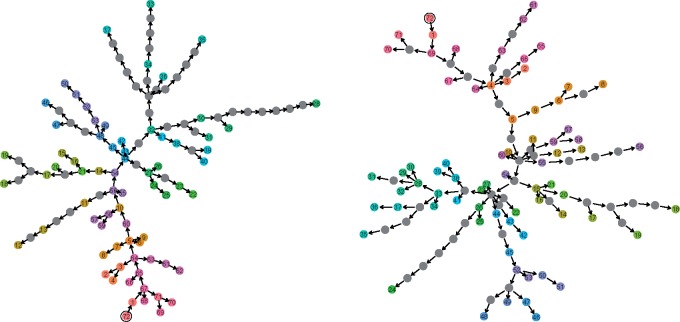
The number of transmission trees versus the number of cherries (top) and the Sackin measure of imbalance (bottom) over 500 random phylogenies each with 20 tips.

For the TransPhylo and empirical STraTUS samples, we show the geometric median transmission trees in figure 10. Although these are not the same, they share a number of transmission events and features. The distribution of unsampled cases is differs notably because STraTUS does not take branch lengths into account in placing them, whereas TransPhylo does.

## Discussion

In this paper, we have explored the mathematics of the set of transmission trees for a known phylogeny, if internal nodes of that phylogeny are not taken to represent infection events, in greater depth and with more rigor than in any previous work. We also give algorithms for uniform sampling of transmission trees. We acknowledge that in most cases a uniform sample from transmission tree space will not be the ideal final tool for inferring epidemic dynamics. However, this work, in addition to establishing a firm footing for further theoretical work of this nature and providing a new means to investigate the relationship between the properties of an epidemic phylogeny and of the epidemic itself, has several other potential applications.

The packages TransPhylo (Didelot et al. 2014, 2017) and BEASTLIER (Hall et al. 2015) both employ MCMC sampling of partitioned trees to estimate transmission trees, for a fixed phylogeny in the former case and a variable one in the latter. The uniform sampling procedure detailed here, perhaps together with metrics on phylogeny and transmission tree space (Kendall and Colijn 2016; Kendall et al. 2018) may prove valuable in the design of improved transition kernels for these algorithms. A uniform sampler for transmission trees may also be useful in a two-stage importance sampling approach of the type employed by Numminen et al. (2014), wherein a uniform sample of transmission trees are sampled, given importance weights according to their likelihood under a model of transmission and then resampled with probability proportional to those weights.

Furthermore, approaches such as TransPhylo, BEASTLIER, phybreak and others make use of a number of models and prior beliefs, such as the nature of the natural history of the pathogen (which is used to inform a likelihood based on time between infection and transmission using a generation time), the sampling fraction and sampling process, and a coalescent model for the within-host pathogen evolution. These parameters are difficult to estimate in any single outbreak data set (particularly in-host evolutionary parameters), and may vary from one outbreak or setting to the next. Reusing past estimates may not solve the problem. The ability to very rapidly sample from all transmission trees consistent with a phylogeny could allow outbreak investigators to quickly get a grip on which putative transmission events are and are not consistent with genomic data, without making strong assumptions on unknown parameters. That the STraTUS sample occupies a larger area of transmission tree space than the TransPhylo sample is presumably a consequence of the uniform sampling approach giving equal probabilities to histories that are outliers according to the TransPhylo model. However, the fact that the TransPhylo set is fully contained in the area covered by the STraTUS set is encouraging. This is true only when the number of unsampled hosts is roughly similar, and hence acquiring an at least reasonably accurate estimate of that number would be advisable.

Perhaps counter-intuitively, we see from figure 8 that unbalanced phylogenies actually admit more transmission trees than balanced ones. This suggests that the fully unbalanced tree (see proposition 3) may be the most flexible phylogeny of all with respect to potential epidemic histories, a potential analytical result that warrants investigation. However, this may be of largely theoretical interest as it ignores branch lengths and hence plausible infection timings. Previous work has shown that the number of potential neighbors for a host in the transmission tree is smaller when the phylogeny is unbalanced (Leventhal et al. 2012), and we do see this pattern when applying time limits to our Yule and unbalanced trees (see fig. 6). The time limits place very useful constraints on the transmission tree set and we recommend their use in STraTUS whenever possible; it should be borne in mind that without them, a transmission tree in which the last-sampled host is the index host is just as probable as any other. The simple cutoff approach to identifying possible infectious periods used here could be refined in further work.

The main assumption in transmission tree inference that we are unable to relax is the complete bottleneck at infection. The partition approach basically requires this, as to discard it is to discard the requirement that the region of a phylogeny associated with each host is connected. Without this, any number of transitions amongst the hosts can occur on any branch, and thus the set of transmission trees is infinite. We would argue that that set is rather less useful than the one we present here, as large numbers of reinfection events will be rare for most pathogens. An approach similar to ours which allows for the transmission of multiple lineages at transmission without conflating that with regular reinfection would be a useful subject for future work. The importance of the bottleneck assumption in practice has not been extensively studied. Consequential violations of connectedness, where transmission trees exist that are actually impossible under the complete bottleneck assumption (see supplementary fig. S3, Supplementary Material online), require not just that multiple lineages be transmitted, but that two or more of them are later either transmitted onwards to different hosts, or sampled. How likely this is to happen in practice will vary from pathogen to pathogen and setting to setting; it is more plausible when the “hosts” in the transmission tree are taken to be geographical locations, which has been a standard approach in agricultural epidemics (Ypma et al. 2013; Hall et al. 2015), rather than when they are individual organisms. It is also unclear whether such an event would ever leave a sufficient signal on the pathogen genome to allow its identification. A family of nonphylogenetic methods to estimate transmission trees that do not make the complete bottleneck assumption has been developed (Worby et al. 2014, 2016), and a parsimony approach, as implemented in, for example, phyloscanner (Wymant et al. 2018), will readily make such reconstructions, but we are not aware of any similar papers to this one examining the interaction between transmission tree space and the phylogeny when the assumption of single lineage transmission is not made.

In summary, we have built on previous work linking transmission trees to partitions of the nodes of a phylogeny to outline procedures by which, for a known tree, possible epidemic histories can be enumerated and sampled from. We also showed how this is possible when the assumptions of complete and single sampling are relaxed. We have presented some examples of how these algorithms can be used to investigate the impact of the phylogeny on the transmission tree, and as a quick alternative to more intensive statistical approaches to the reconstruction of the latter. Future work may refine the handling of infectious periods and unsampled cases, or employ this sampler as a component of a more sophisticated statistical approach.

## Materials and Methods

The Yule and biased trees were generated using the rtreeshape function in apTreeshape v1.5 (Bortolussi et al. 2006). The random phylogenetic trees used to investigate the relationship between transmission tree count and other statistics were generated using the rtree function in ape v1.5 (Paradis and Schliep 2019). All trees were visualized with ggtree v3.7 (Yu et al. 2017). Transmission trees were compared using the metric of Kendall et al. (2018) implemented in treespace 1.1.3 (Jombart et al. 2017). Principal component analysis was performed using ade4 v1.7-13 (Chessel et al. 2004). Sequencing, alignment and BEAST analysis of the *Mycobacterium tubercolosis* data set has been previously described (Roetzer et al. 2013; Didelot et al. 2017).

## Supplementary Material

Supplementary data are available at *Molecular Biology and Evolution* online.

## Supplementary Material

Supplementary_Material_msz058
