# Supplementary material for: Transmission Trees on a Known Pathogen Phylogeny: Enumeration and Sampling
Source: Mol Biol Evol. 2019 Mar 14;36(6):1333–43. doi: 10.1093/molbev/msz058 (PMC6526902; doi:10.1093/molbev/msz058)
Supplement: Supplementary_Material_msz058 [file supplementary_material_msz058.zip › SuppFigures.pdf]

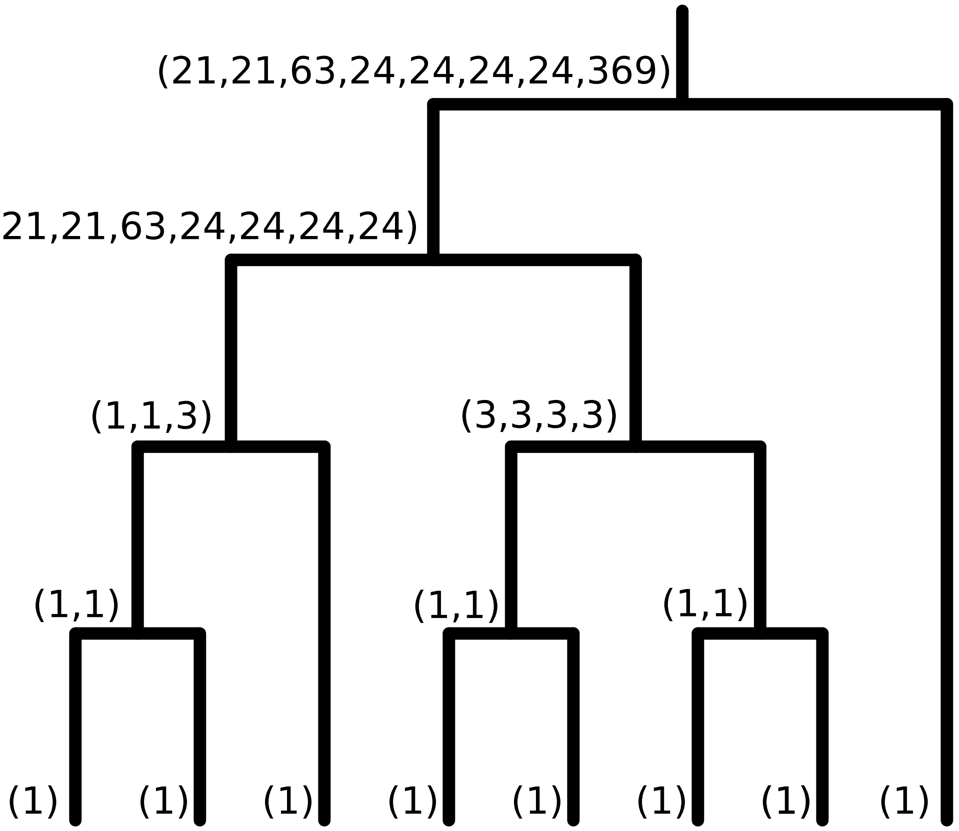

Figure S1: The calculation of all $|\mathbf{P}^i(\mathcal{T})|$ if $\mathcal{T}$ is the tree in figure 4. Each internal node u rooting a subtree $\mathcal{T}_u$ is annotated with a tuple of the nonzero values of $|\mathbf{P}^i(\mathcal{T}_u)|$, appearing in the same order as the subtree's tips as displayed from left to right.

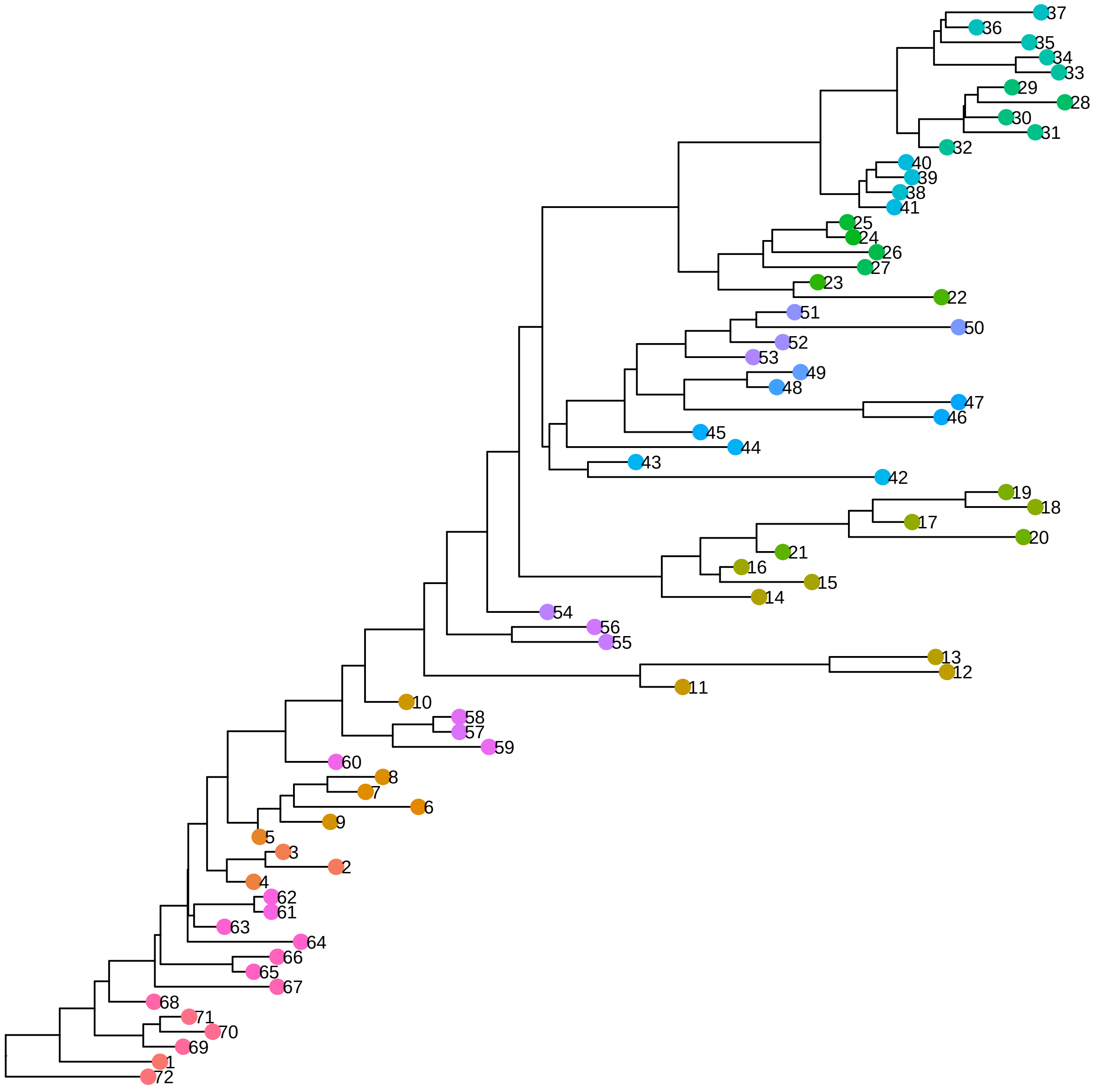

Figure S2: The 72-tip phylogeny of TB isolates used to compare *TransPhylo* and *STraTUS*. Colours and numbers are the same as those in figure 10.

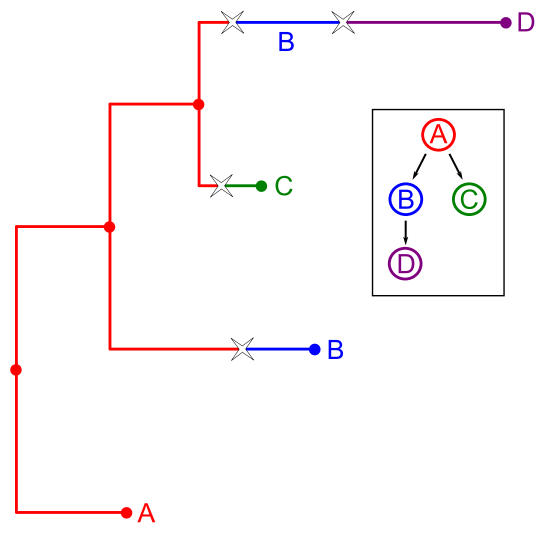

Figure S3: For this phylogeny, the inset transmission tree is impossible under the complete bottleneck assumption, but possible if it is relaxed and host A can transmit two lineages to host B, as shown. Stars represent transmission events.

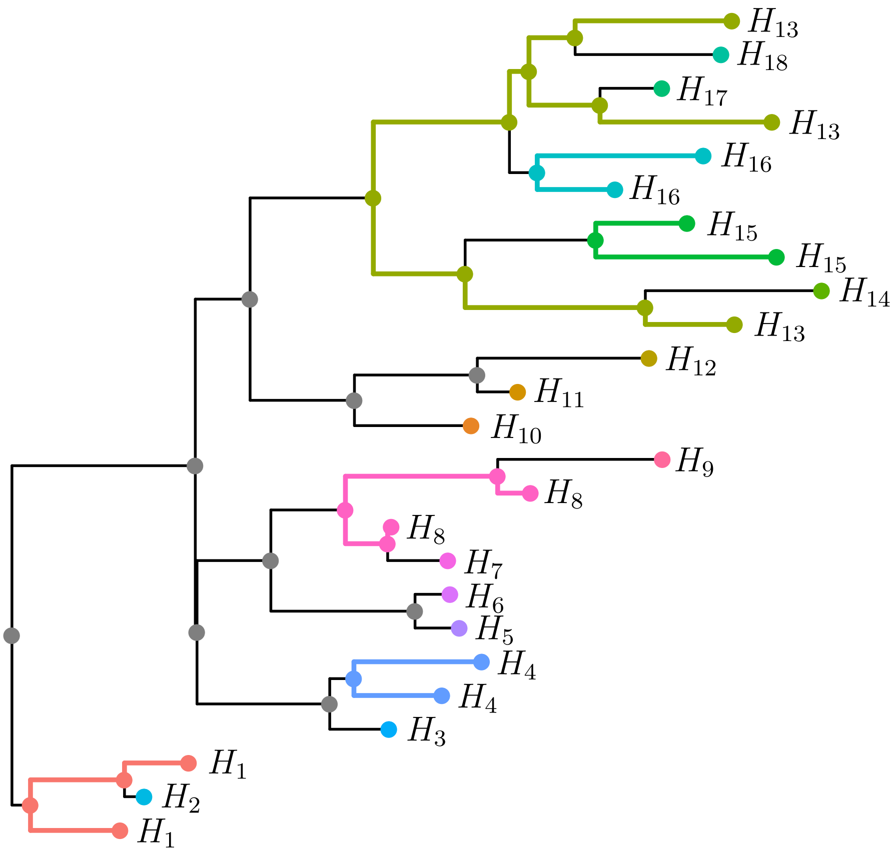

Figure S4: An illustration of bridges. The partition $\mathfrak{H}$ of the tips of this tree has eighteen parts $\{H_1, \ldots, H_{18}\}$, and each tip is labelled, and coloured, according to the part it belongs to. Six parts (H_1, H_4, H_8, H_{13}, H_{15} and H_{16}) contain more than one tip, and define bridges that contain internal nodes. For each such H_i the nodes in the bridge $\mathbf{b}(H_i)$, and the branches connecting them, appear as the same colour as the elements of H_i. Nodes that are not members of any bridge are grey.
